# Complete Genome Sequence of a Third- and Fourth-Generation Cephalosporin-Resistant Comamonas kerstersii Isolate

**DOI:** 10.1128/MRA.00391-21

**Published:** 2021-07-15

**Authors:** Aline I. Moser, Edgar I. Campos-Madueno, Peter M. Keller, Andrea Endimiani

**Affiliations:** a Institute for Infectious Diseases (IFIK), University of Bern, Bern, Switzerland; Montana State University

## Abstract

Here, we report the complete genome sequence of Comamonas kerstersii 3132976, a strain isolated from a human rectal swab sample in Switzerland. The isolate was resistant to third- and fourth-generation cephalosporins and possessed a novel class A β-lactamase gene. The complete genome is 3,693,404 bp long with a GC content of 59.4%.

## ANNOUNCEMENT

Comamonas kerstersii is a nonfermenting pathogen sporadically associated with appendicitis, urinary tract infections, psoas abscess, and salpingitis (1–5); it can also be detected in stool (1, 4). Several *C. kerstersii* isolates resistant to third-generation (e.g., ceftazidime and cefotaxime) and fourth-generation (e.g., cefepime) cephalosporins were reported, but their genome sequences are not available (1, 2).

In December 2019, a Swiss man in his 70s returning from Croatia was admitted at the Inselspital (Bern, Switzerland). *C. kerstersii* strain 3132976 was isolated from a rectal swab that was plated onto a CHROMagar ESBL plate and incubated at 37°C overnight. Species identification was achieved by matrix-assisted laser desorption ionization–time of flight mass spectrometry (MALDI-TOF MS) (Bruker) and later confirmed using the sequenced genome and the Type (strain) Genome Server (https://tygs.dsmz.de/). Phenotypic testing performed using the microdilution GNX2F and ESB1F Sensititre panels (Thermo Fisher Scientific) indicated that the strain was fully susceptible to carbapenems, aminoglycosides, tetracyclines, fluoroquinolones, and polymyxins but had a phenotype consistent with the production of an extended-spectrum β-lactamase (resistant to ceftazidime, cefotaxime, and cefepime but susceptible to either ceftazidime or cefotaxime combined with clavulanate) (6).

Genomic DNA was obtained from a fresh overnight culture grown on a MacConkey agar plate at 37°C using the PureLink microbiome DNA purification kit (Thermo Fisher Scientific). Whole-genome sequencing (WGS) was performed by combining the NovaSeq 6000 platform (NEBNext Ultra II DNA library prep kit for Illumina; 2 × 150-bp paired-end reads) and the MinION device (SQK-RBK004 library; FLO-MIN 106D R9 flow cell; Oxford Nanopore). Adapters from the Illumina and Nanopore reads were removed with Trimmomatic v0.36 and Porechop v0.2.4, respectively (7, 8). The Nanopore reads were used to generate a *de novo* assembly with Flye v2.7-b1585 (parameters: - -nano-raw, - -genome-size 3.7m). The resulting circular assembly was polished with the trimmed Illumina reads using Pilon v1.22 (9, 10). Gene annotation was performed using the NCBI Prokaryotic Genome Annotation Pipeline (11). The quality of the assembly was assessed using CheckM v1.1.2 (12). The final genome was analyzed using the Center of Genomic Epidemiology services (www.genomicepidemiology.org/), IslandViewer 4, and PHASTER to identify antimicrobial resistance genes (ARGs) and horizontal gene transfer regions (13, 14). Default parameters were used for all software unless otherwise specified.

**FIG 1:**
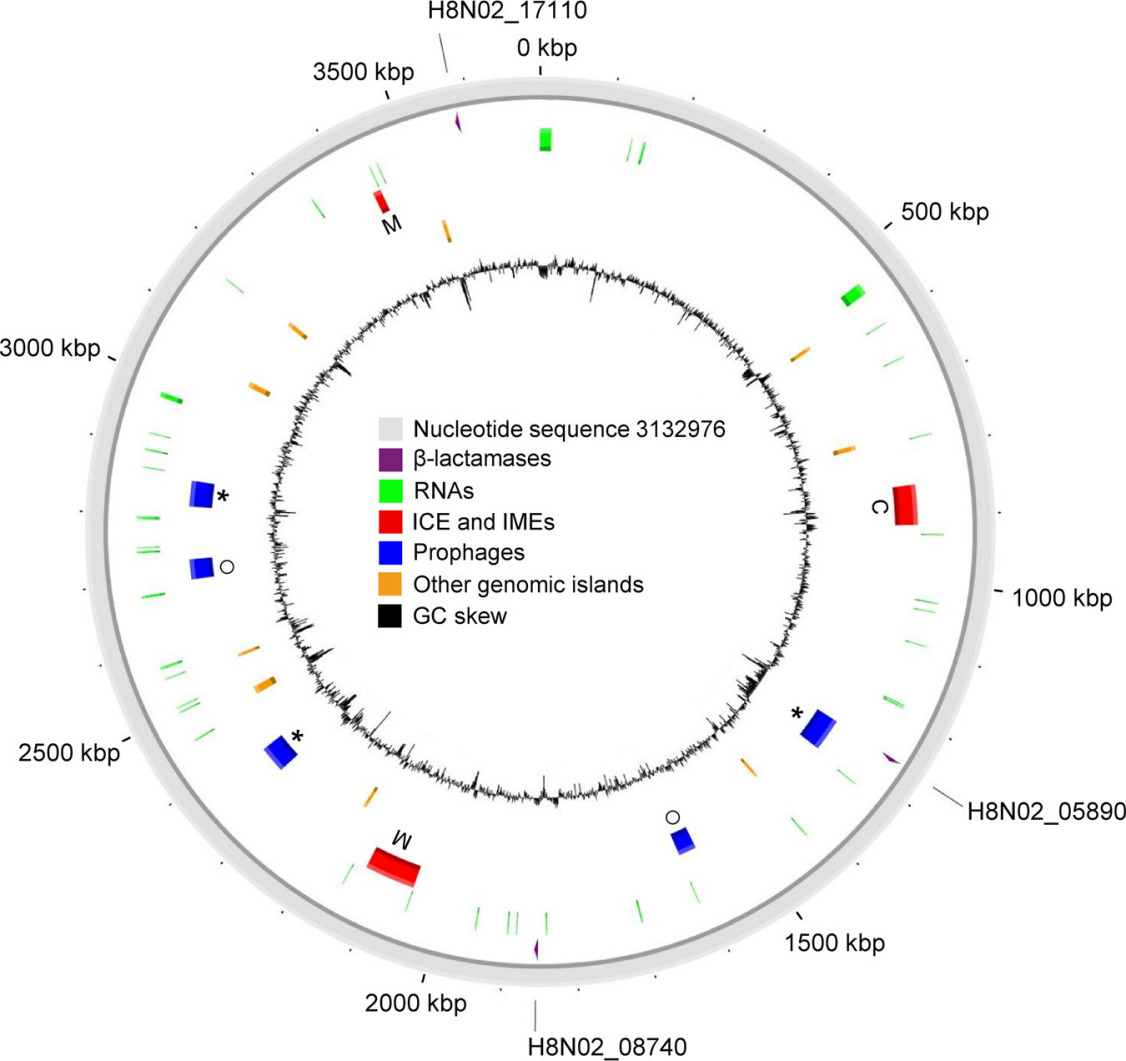
Genome map of *C. kerstersii* strain 3132976. Predicted elements in the genome of strain 3132976 (outermost circle) are presented in different colors. M indicates predicted integrative and mobilizable elements (IMEs), C indicates the predicted integrative and conjugative element (ICE), the circle indicates intact prophage regions, and the asterisk indicates incomplete prophage regions. The β-lactamases are depicted as arrows.

The sequencing generated a total of 41,099 Nanopore (*N*_50_, 8,006 bp) and 15,382,964 Illumina reads. The assembled circular genome was 3,693,404 bp long with a coverage depth of 293× and a GC content of 59.4%. Aligning an independent Illumina short read assembly with SPAdes (v3.14) (data not shown) and Illumina short read mapping to the complete hybrid genome showed contig and short read sequence overlap at the start and end junctions of the final genome, indicating that it was indeed circular (15). Annotation identified 3,337 coding DNA sequences (CDSs), 105 tRNAs, and 22 rRNAs. A total of 17 regions of probable horizontal origin were identified. Except for three β-lactamases (CDSs H8N02_05890, H8N02_08740, and H8N02_17110), no further ARGs were identified (Fig. 1). H8N02_17110 and H8N02_08740 (class A and C β-lactamases, respectively) are present in all seven available C. *kerstersii* genomes (16). In contrast, the class A β-lactamase H8N02_05890, located within a predicted prophage, was different from any publicly available sequence and showed the best amino acid homology (79.6%) with a β-lactamase from Comamonas terrigena (GenBank accession no. WP_183302591). The biochemical profile of this novel class A β-lactamase should be investigated.

### Data availability.

The complete hybrid genome sequence of *C. kerstersii* 3132976 is available in GenBank (CP060413) under BioProject PRJNA657966. The raw reads were deposited in the Sequence Read Archive (SRA) under SRR14226838 and SRR14226837 for Illumina and Nanopore reads, respectively.
